# Immunomodulatory Effects of Fluoroquinolones in Community-Acquired Pneumonia-Associated Acute Respiratory Distress Syndrome

**DOI:** 10.3390/biomedicines12040761

**Published:** 2024-03-29

**Authors:** Resti Yudhawati, Nisrina Fitriyanti Wicaksono

**Affiliations:** 1 Department of Pulmonology and Respiratory Medicine, Faculty of Medicine, Universitas Airlangga, Surabaya 60132, Indonesia; 2 Department of Pulmonology and Respiratory Medicine, Universitas Airlangga Teaching Hospital, Surabaya 60015, Indonesia; 3 Department of Pulmonology and Respiratory Medicine, Dr. Soetomo General Hospital, Surabaya 60286, Indonesia; 4 Faculty of Medicine, Universitas Airlangga, Surabaya 60132, Indonesia; nisrinafitriw15@gmail.com

**Keywords:** immunomodulatory, fluoroquinolones, community-acquired pneumonia, acute respiratory distress syndrome

## Abstract

Community-acquired pneumonia is reported as one of the infectious diseases that leads to the development of acute respiratory distress syndrome. The innate immune system is the first line of defence against microbial invasion; however, its dysregulation during infection, resulting in an increased pathogen load, stimulates the over-secretion of chemokines and pro-inflammatory cytokines. This phenomenon causes damage to the epithelial–endothelial barrier of the pulmonary alveoli and the leakage of the intravascular protein into the alveolar lumen. Fluoroquinolones are synthetic antimicrobial agents with immunomodulatory properties that can inhibit bacterial proliferation as well as exhibit anti-inflammatory activities. It has been demonstrated that the structure of fluoroquinolones, particularly those with a cyclopropyl group, exerts immunomodulatory effects. Its capability to inhibit phosphodiesterase activity leads to the accumulation of intracellular cAMP, which subsequently enhances PKA activity, resulting in the inhibition of transcriptional factor NF-κB and the activation of CREB. Another mechanism reported is the inhibition of TLR and ERK signalling pathways. Although the sequence of events has not been completely understood, significant progress has been made in comprehending the specific mechanisms underlying the immunomodulatory effects of fluoroquinolones. Here, we review the indirect immunomodulatory effects of FQs as an alternative to empirical therapy in patients diagnosed with community-acquired pneumonia.

## 1. Introduction

Community-acquired pneumonia (CAP) is one of the most common infectious diseases, contributing significantly to reported rates of mortality and morbidity around the world [1,2]. Pathogens that cause CAP are classified into two types: ‘typical’ agents, including Gram-positive organisms (such as *Streptococcus pneumoniae*, *Staphylococcus aureus*, *Haemophilus influenza*. Group A *Streptococcus* spp.; anaerobes), and Gram-negative organisms (such as *Klebsiella pneumoniae*, *Streptococcus pyogenes*, *Pseudomonas aeruginosa*, *Escherichia coli*, *Acinetobacter baumannii* and *Stenotrophomonas maltophilia*); ‘atypical’ agents include *Legionella pneumophila*, *Mycoplasma pneumoniae*, *Chlamydophila pneumoniae*, *Chlamydophila psittaci*, influenza viruses (A, B), severe acute respiratory syndrome coronavirus 2 (SARS-CoV-2), and other respiratory viruses [3,4,5]. Co-infection with bacteria is a frequent phenomenon observed in respiratory viral infections, leading to an elevation in both morbidity and mortality rates [6,7]. Co-infection, also commonly referred to as “superinfection”, is frequently found during the pandemic of viruses [8,9,10].

Complications may arise as a result of pneumonia, leading to the development of acute lung injury (ALI)/acute respiratory distress syndrome (ARDS): a condition that is associated with significant rates of morbidity and mortality [11,12]. Pneumonia and sepsis are globally recognised as the main risk factor (~75% of cases) for ARDS [13]. A quantitative model study generated data from 13 countries across the world and concluded that ~22.15% of pneumonia patients developed ARDS [14].

The innate immune system serves as the first barrier of defence in the host’s response to pathogens by identifying their pathogen-associated molecular patterns (PAMPs) or microbe-associated molecular patterns (MAMPs). However, during infection, the innate immune response experiences dysregulation, aggravating the severity of illness by increasing the pathogen load as the consequence of inefficient pathogen clearance or irreversibly damaging the organs of patients with sepsis, who subsequently may die as a result of multi-organ failure [15,16]. The innate immune response specific to a particular organ determines the infection severity. The lungs exhibit a robust innate immune response during localised lung infections associated with severe ALI or ARDS, which significantly influences the outcome of the disease [15,17].

The administration of anti-inflammatory agents such as steroids to patients with CAP (with or without shock) remains controversial, although many studies have demonstrated a significant decrease in hospitalisation duration and time to reach clinical stability [18,19,20,21]. Patients with significant inflammatory responses, such as those with a high level of c-reactive proteins (CRPs), may constitute a subset of severe CAP patients who benefit from such corticosteroid therapy, according to accumulated published data [19]. While many studies suggest the benefit of steroids, one study showed increased mortality [22]. Another study also suggested that corticosteroid treatment did not improve survival in CAP patients, while nosocomial infections were increased [23].

Fluoroquinolones (FQs) are a class of synthetic antimicrobial agents that inhibit DNA synthesis by targeting DNA gyrase and topoisomerase IV enzymes [24]. One of FQ’s properties are its broad spectrum; therefore, these compounds are highly active in combating Gram-positive and Gram-negative bacteria, and even anaerobes, mycobacteria and atypical pathogens [24,25,26,27]. Besides the above-mentioned properties, these drugs have been reported to exert anti-oxidative effects both in vitro and in vivo [28,29]. In addition, FQs is also reported to block pro-inflammatory cytokines and chemokines, leading to the disruption of neutrophil chemotaxis [25]. According to the most recent guidelines and the literature available, FQs have been accurately proven to exert immunomodulatory effects, which are clinically advantageous for the treatment of CAP. Immunomodulatory effects in FQs have been described as beneficial to reducing lung damage due to bacterial, viral, and fungal infections in animal models [27,29,30,31,32,33].

The main obstacle to improving the outcome of CAP is the excessive pro-inflammatory response [34]. Several therapeutic options have been tested to improve the outcome of CAP using different strategies. FQs have immunomodulatory effects beyond their antibacterial effects that might be beneficial for patients with CAP. Hence, the present review article focuses on the indirect immunomodulatory effects of FQs, in addition to their direct antibacterial effects, which have been utilised as an alternative to empirical therapy in patients diagnosed with CAP.

## 2. Immunopathogenesis of Pneumonia-Associated ALI/ARDS

ALI/ARDS is a condition that results from heterogeneous aetiologies, with bacterial pneumonia being the dominant cause [11,35]. The disruption in the blood–air barrier due to the infiltration of innate immune cells, the release of inflammatory mediators, and other injury pathways leads to further lung damage and the influx of protein-rich pulmonary oedema [25,36].

Bacteria, both Gram-positive and Gram-negative, viruses, and fungi have uniform molecular patterns known as PAMPs, which are identified by pattern recognition receptors (PRRs) on the surface of the organism’s immune cells, one of which is Toll-like receptor (TLR), before finally binding to them [17,37]. Following the successful entrance of bacteria to the lower airway, bacteria interact with macrophages—PRRs—of the pulmonary innate immune system via their cell walls and intracellular components (lipoteichoic acid, peptidoglycan, nucleic acids, pneumolysin, and other pore-forming toxins), and consequently, transcription factors, such as nuclear factor κappa-B (NF-κB) are activated [17,25,38].

NF-κB represents a family of five transcription factors that play crucial roles in various biological processes that support aspects of differentiation and development, immune response modulation, cell growth, proliferation, apoptosis, and phenotypic outcomes associated with inflammation [39,40]. Notably conserved in all mammalian cells, NF-κB plays a pivotal role in the transcription of genes encoding numerous cytokines and chemokines, including those with pro-inflammatory properties [41]. NF-κB is bound to the inhibitory protein, inhibitory κappa B (IκB), and retained in the cytoplasm of the resting, non-stimulated cell. NF-κB proteins are commonly activated and released in response to various extracellular ligands, including agents that induce a DNA damage response (DDR), leading to the translocation of DNA-binding protein dimers to the nucleus after dissociating from IκB molecules [39,40,42].

Signal transduction pathways triggered by cell stimulation with multiple agonists initiate the activation of IκB kinases (IKK). IKK induces the phosphorylation of IκB, which is subsequently followed by a rapid degradation of the IκB proteins, leading to the liberation of NF-κB. Consequently, NF-κB translocates into the nucleus, where it binds to DNA and initiates the process of transcriptional activation [43]. The nuclear translocation of the activated transcription factor triggers the induction of genes encoding various pro-inflammatory cytokines (interleukin (IL)-1β, IL-6, IL-8, IL-17, IL-18, tumour necrosis factor (TNF)) and chemokines (CCL8, monocyte chemotactic protein 1 (MCP-1), macrophage inflammatory protein 1α (MIP-1α)) [38,44]. High levels of cytokines and chemokines in plasma and bronchoalveolar lavage (BAL) fluids are related to poor clinical outcomes in ARDS, including a high mortality rate [44,45]. A study observing ARDS patients showed that Staphylococcus, Streptococcus, and Enterobacteriaceae were identified as the specific bacteria associated with elevated levels of IL-6 in non-surviving patients with ARDS. Streptococcus secretes pneumolysin and MUC5B, both of which have been found to be closely associated with lung cell fibrosis and lung inflammation [46].

The activation of protein-1 (AP-1) is observed to have a role in ALI/ARDS pathogenesis by trans-activating pro-inflammatory cytokines and other genes that lead to lung damage [47]. In response to various stimuli, TLR4 induces and recruits intracellular adaptor proteins, resulting in a signalling cascade that involves similar signalling molecules to NF-κB signalling on the TLR4/TRAF6 axis [48,49]. TRAF6 activates TAK1 (transforming growth factor-activated kinase 1) and subsequently initiates a MAPK (mitogen-activated protein kinase) cascade that includes ERK (extracellular signal-regulated kinases), JNK (c-Jun N-terminal kinases), and p38, leading to the activation and nuclear translocation of AP-1. The activated AP-1 interacts with the promoters of pro-inflammatory cytokines, increasing their expression [47,48].

p38MAPK, an essential signalling protein, has been established in the literature to play a major pro-inflammatory role in the development of ARDS at both the transcriptional and post-transcriptional levels. Evidence has suggested that the activation of p38MAPK plays a critical role in the synthesis of inflammatory cytokines [50]. Among its four isoforms, p38α MAPK was the first to be identified for its function in the regulation of pro-inflammatory cytokines. IL-8 and IL-6 production in response to IL-1 and TNF-α, respectively, were then associated with p38α MAPK. As the initiation factors, IL-1β and TNF-α have the ability to directly injure vascular endothelial cells as well as activate a series of effector cells [51].

Following damage-associated molecular pattern molecules (DAMPs) or alarmins derived from the host, which further bind with TLR on the lung epithelium and alveolar macrophages, the polarisation of alveolar macrophages (AMs), neutrophil extracellular trap (NET)osis, the pro-inflammatory response exhibited by T helper 17 subsets, and the anti-inflammatory and regenerative functions performed by T regulatory cell subsets occur [52,53,54]. During infections, DAMPs and PAMPs synergistically stimulate the synthesis and secretion of pro-inflammatory cytokines and chemokines, as well as induce cell differentiation and cell death [52,53]. Pro-inflammatory cytokines have double effects on the host defence mechanisms; on the one hand, they promote the activation of adaptive immunity that releases multiple mediators such as prostaglandins, leukotrienes, and proteases; however, on the other hand, they induce direct and indirect injury to the microvasculature of the host [37].

The secreted pro-inflammatory cytokines stimulate the activation of localised vascular endothelia cells; while the release of chemo-attractants attracts more monocytes and neutrophils, as well as the exudation of pro-inflammatory complement proteins and acute-phase reactants [38]. The recruitment of neutrophils is a defining feature of ARDS and is considered to play a critical role in the course of the disease [55]. When the neutrophils migrate to the epithelium, these cells elicit toxic mediators, including proteases, nitric oxide (NO), reactive oxygen species (ROS) and NET, which are essential host defence mechanisms [32,56]. Neutrophils release extracellular fibres, myeloperoxidase, DNA, and neutrophil elastase into the extracellular environment during pathogen invasion as a defensive mechanism, referred to as NETosis, to create a network for microbe entrapment. However, excessive neutrophil activation and unbalanced inflammatory responses may result in further tissue damage, including endothelial and epithelial damage, which may subsequently lead to an elevation in the permeability of these cells [55].

The increase in endothelial and epithelial permeability enables the transmigration of leucocytes and ultimately results in the influx of oedematous fluid and red blood cells (RBCs). RBCs produce cell-free haemoglobin, which aggravates damage through oxidant-dependent pathways. The airspace is filled with oedematous fluid, subsequently resulting in impaired gas exchange and profound hypoxemia. Vascular injury and alveolar oedema also participate in the decrease in CO_2_’s excretion ability (hypercapnia), causing an increase in pulmonary dead space in ARDS. Furthermore, hypoxaemia and hypercapnia reduce alveolar oedema clearance by impairing vectorial sodium transport [45,55].

**Figure 1:**
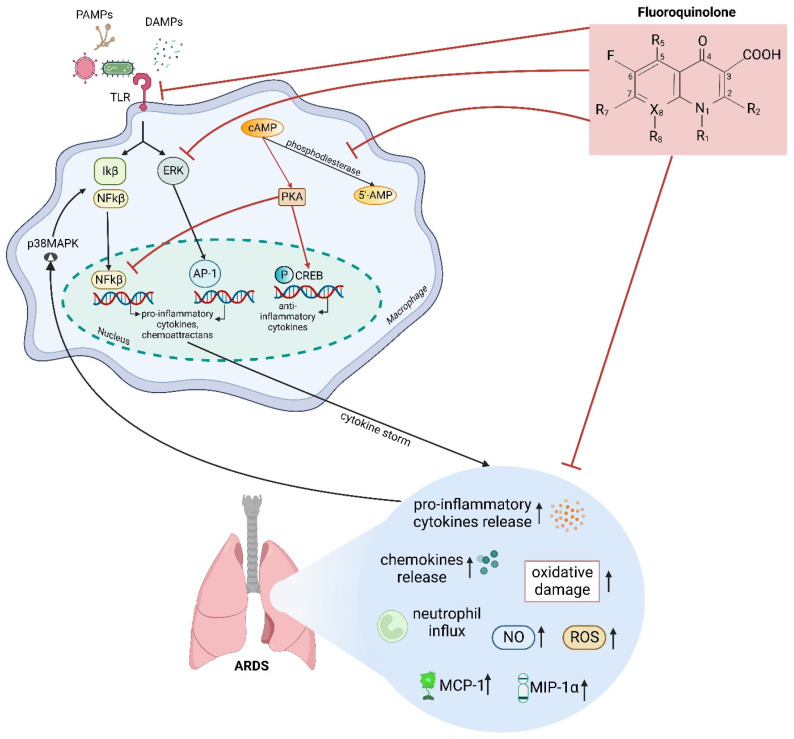
(**Black arrow**) PAMPs of microorganisms and DAMPs released by the infected or injured cells interact with PRRs on the surface of the organism’s immune cells, one of which is TLR. The stimulation of the macrophage TLR receptor rapidly activates not only the NF-κB pathway but also the ERK pathway. The TLR-activated ERK pathway regulates AP-1 transcriptional activity. The nuclear translocation of the activated transcription factor (NF-κB and AP-1) triggers the induction of genes encoding various pro-inflammatory cytokines (IL-1β, IL-6, TNF-α, etc.)/chemokines (MCP-1, MIP-α, etc.) which stimulate the transepithelial migration of neutrophils, oxidative damage, and increases in NO and ROS. All of these mechanisms result in a cytokine storm and then subsequently lead to ARDS. Pro-inflammatory cytokines and environmental stress cause p38MAPK activation, which plays a role in the regulation of the transcriptional activity of NF-κB. (**red arrow**) Fluoroquinolones exert their immunomodulatory activity by inhibiting the TLR and ERK signalling pathways. FQs also inhibit the activity of phosphodiesterase activity and result in the accumulation of intracellular levels of cAMP. The accumulation of cAMP levels leads to an augmentation in the activity of PKA, which is known to inhibit the transcription factor of NF-κB, thereby inhibiting further lung damage by reducing pro-inflammatory cytokines and chemokine production, neutrophil influx, oxidative damage, as well as NO and ROS. In addition, PKA, in turn, activate CREB as a primary regulator of anti-inflammatory and immune response. We created this figure using the BioRender online app and license.

Figure 1 illustrates the immunopathogenesis of ARDS (black arrow).

## 3. Specific Features of Quinolone Molecules Related to Immunomodulatory Effects

Quinolone is a class of antibiotics with a bicyclic structure derived from the 4-quinolone compound [57]. The carboxylic acid group at position 3 and the carbonyl at position 4 appear to have a significant role in determining the activity of quinolones. In addition, bulky substituents on one face of the bicyclic core are permitted, specifically at positions 1 and 7 and/or 8, and they are likely important in determining the spectrum of quinolone antibiotics. Regarding these substituents, most quinolones can be classified into three primary types based on their sidechains: piperazinyl-, pyrrolidinyl-, and piperidinyl-types [58]. A class of 6-fluoro-7-piperazinyl-4-quinolones, or fluoroquinolones, are synthetic antimicrobial agents that have a wide range of activity. These agents are derived from quinolones and have a fluorine atom attached to the central ring [59].

FQs have indirect antibacterial effects in addition to their intrinsic antibacterial activity, which may be due to their immunomodulatory activity [41]. Based on their pharmacokinetic profile and antimicrobial activity, fluoroquinolones are classified into four generations. Nalidixic acid was the first within the quinolone class that was discovered to have antibacterial activity. The first generation of quinolones was retracted from the market shortly after their introduction. Besides nalidixic acid, the first generation included cinoxacin [60]. The second generation of quinolones began with the formation of fluoroquinolones by fluoridating the quinolone molecule at position C6, which enhanced the compounds’ activity against Gram-negative bacteria. The addition of a cyclopropyl group at position R1 further improved the compounds’ overall activity [61,62]. Examples of second-generation drugs include ciprofloxacin, enoxacin, norfloxacin, ofloxacin and lomefloxacin. However, lomefloxacine was withdrawn from the market after a few years of approval for clinical use [63]. The third generation of quinolones was initiated with the development of fleroxacin. In this generation, more powerful FQs, including levofloxacin, sparfloxacin, grepafloxacin and gatifloxacin, were developed [64]. According to the WHO’s list of essential medications, ciprofloxacin and levofloxacin are the most commonly prescribed drugs [65]. Third-generation compounds also have an additional incorporation of new substituents, namely a chloro group (Cl) at the R8 position, which demonstrates enhanced bactericidal action against Gram-positive bacteria and atypical bacteria [62]. In the current market, sparfloxacin, gatifloxacin, and grepafloxacin were discontinued for clinical use [63]. The specific insertion of a cyclic diamine piperazine molecule at position C-7 and a fluorine atom at position C-6 have synthesised present-day FQs, which exhibit notable efficacy against anaerobic, Gram-positive, and Gram-negative bacteria. The third and fourth generations of FQs contain a methoxy group at the C-8 position and are currently licensed for the treatment of respiratory tract infections, including lethal pulmonary tuberculosis [66]. The fourth generation is represented by trovafloxacin, moxifloxacin, and gemifloxacin, with trovafloxacin being withdrawn from the market [63]. Some FQs were withdrawn from the market after a few years of approval for use due to an increased risk of various severe adverse effects that were associated with FQ administration [67,68].

FQs were initially optimised and developed as antimicrobial agents, and each generation appeared to confer increased potency. However, according to a review article by Anderson and Osheroff [69], multiple reports have indicated that their capabilities may extend beyond antimicrobial effects only. The extensive utilisation of quinolone derivatives in clinical applications has contributed to the identification of their immunomodulatory effects [70].

Several studies have reported how certain FQs exhibit in vitro anti-proliferative properties through various mechanisms, including the induction of apoptosis, the disruption of the biochemical transformation of potentially cancerous cells, the enhancement of other chemotherapeutic agents’ uptake, and/or mediation of immunomodulatory responses [71,72,73]. Quinolones, in general, exert their modulating effects only when combined with a co-stimulant. Nevertheless, it has been widely observed that quinolone compounds have a tendency to reduce the production of pro-inflammatory cytokines. The induction of cytokines has been observed only in a subset of FQs, and this effect seems to be related to the presence of the cyclopropyl moiety at the N1 position [74].

Quinolones are comprised a bicyclic ring structure with a substitution at position N-1 containing various moieties. The majority of current agents contain a fluorine atom at position 6 and a nitrogen heterocycle moiety at position C7 [75]. The precise mechanism remains unclear, but it is conceivable that certain FQs may activate transcription factors, such as AP-1, which are known to be associated with elevated cytokine levels [76]. The observed variations in outcomes across multiple studies can be attributed to the distinct chemical structures and different pathogens involved. The outcomes of these studies were likely influenced by the timing and frequency of administered doses [31,32,77].

**Figure 2:**
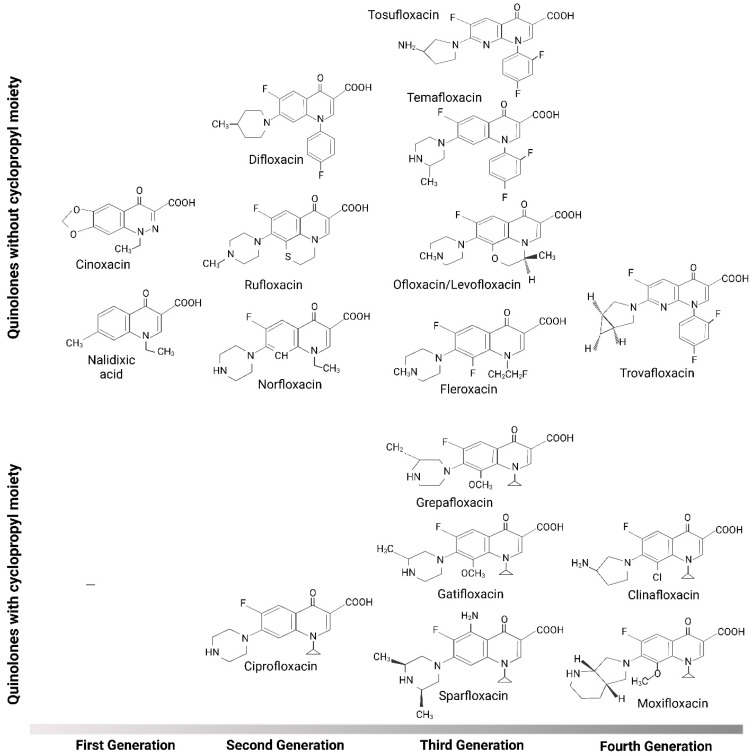
Structure of fluoroquinolones with and without cyclopropyl moiety. We created this figure using the BioRender online app and license.

Immunomodulatory effects are especially evident in FQs with a cyclopropyl-moiety at position N1 of their quinolone ring, such as ciprofloxacin and moxifloxacin [78]. Substituents located at position 1 of the basic quinolone structure affect antibacterial activity potency. The presence of a cyclopropyl substituent at this specific position, which is present on all of the new FQs besides levofloxacin and trovafloxacin, is regarded as the most optimal for activity [79]. Several quinolones with and without the cyclopropyl group on N1 are presented in Figure 2.

The presence of a cyclopropyl group at position N1 of the quinolone molecule has been shown to exhibit superior antibacterial action against Gram-negative bacteria, as demonstrated by Chu et al. [80] and Domagala [81]. Exposure to ciprofloxacin and CP-115,953, which contain a cyclopropyl group at position N1, significantly boosted the release of interferon (IFN)-y from human peripheral blood cells in vitro compared to compounds without this moiety [82,83]. Similarly, certain structural properties of FQs are also associated with these agents’ non-antibacterial activity against eucaryotic topoisomerase II. The study conducted by Yamashita et al. [84] presented the augmented anti-leukemic effects observed in a murine leukaemia model, which were accompanied by an increased inhibitory activity against topoisomerase II. This enhanced activity was observed specifically in quinolones that possessed the same cyclopropyl group attached to the N1 position of the quinolone ring. A comparable discovery was documented in a comparative study examining the effect of six quinolones on the production of IL-3 and the granulocyte–macrophage colony-stimulating factor (GM-CSF) by stimulated murine splenocytes [74]. Under the same experimental conditions, the production of these aforementioned cytokines was found to be enhanced exclusively by quinolones that incorporated the cyclopropyl group at position N1. Conversely, quinolones lacking in this moiety had either no impact or an inhibitory effect. The data presented in these studies provide clear evidence that specific FQs containing an N1-cyclopropyl group exhibit immunomodulatory effects [78].

Another study revealed that gemifloxacin, a fourth-generation fluoroquinolone drug, has significant immunomodulatory potential. Due to the presence of a cyclopropyl N-1 group in its structure, gemifloxacin may have the potential to influence both innate and adaptive immune systems. This drug has demonstrated a dual effect on both innate and adaptive immune systems, whereby it enhances the activity of the innate system while suppressing the adaptive immune system. The humoral immune response is produced through the synthesis of antibodies that target the specific epitope of the antigen employed to induce the immune response [85].

## 4. Immunomodulatory Activity of Fluoroquinolone in ARDS

The immunomodulatory effects of quinolones are mostly anti-inflammatory and have been widely documented in in vitro models; however, the precise mechanisms by which quinolones act as immunomodulators are not yet fully comprehended [65,86,87]. FQs are suggested to exhibit their immunomodulatory activities via influencing phosphodiesterase activity and promoting the production of intracellular cyclic adenosine monophosphate (cAMP) [88,89]. Presumably, quinolones elevate intracellular levels of cAMP by inhibiting the activity of phosphodiesterase enzymes [90]. cAMP is essential for the regulation of various inflammatory responses in innate immune cells [91]. The intracellular levels of these cyclic nucleotides are primarily regulated by enzymes called phosphodiesterases (PDEs), which catalyse the hydrolysis of a cyclic phosphate bond in cAMP and cyclic guanosine monophosphate (cGMP) to produce the inactive 5′-AMP and 5′-GMP [92]. The predominant subtype of PDE in neutrophils is PDE4, which is involved in the pathogenesis of inflammatory diseases [93]. Bailly et al. suggested that the inhibitory effect of ciprofloxacin on TNF-α and IL-1 may possibly be attributed to its inhibitory effect on phosphodiesterase, resulting in the accumulation of intracellular cAMP [94]. The accumulation of cAMP levels leads to an augmentation in the activity of protein kinase A (PKA), which is known to decrease the TNF-α expression [92]. Several investigations have indicated that this accumulation inhibits TNF-α and IL-1 production in mononuclear phagocytes [94,95,96]. A study conducted by Blaine et al. [97] provided evidence of the phosphodiesterase inhibitory effect of ciprofloxacin, which caused cAMP to accumulate in the cells and increase PKA activity, which, in turn, is known to inhibit the production of TNF-α in stimulated monocytes. There is a proposition that cAMP, which functions as a second messenger, has the potential to function as an anti-inflammatory agent through its ability to stimulate downstream signalling pathways, including PKA and the exchange protein directly activated by cAMP (Epac), while also reducing the secretion of cytokines [88]. PKA then subsequently activates CREB (cAMP response element-binding protein), which is a primary regulator of anti-inflammatory and immune response [98]. Furthermore, recent results present compelling evidence suggesting that certain cellular properties associated with cell motility can also be regulated by modulating cAMP levels [99,100].

The mechanisms by which quinolones exert their effects on various cytokines and chemokines involve the regulation of certain key cellular transcription factors. The transcription factor known as NF-κB is one of the key factors in cellular signals [41,42]. It is crucial to emphasise that the modulatory effects of quinolones are not observed when they are used alone, and an extra stimulating effect is required. The stimulators independently induce an intracellular stress response, such as lipopolysaccharide, which may interact synergistically with the inhibitory effects of topoisomerase-II to induce the augmented immunomodulatory event [41]. The inhibition of topoisomerase II impacts protein C kinase, resulting in enhanced AP-1 activity, which has been associated with increased cytokine levels [101,102]. The precise sequence of events that underlie the effect of quinolones on the above transcription factors and their activators, including Iκβ and potentially IKK, remains unknown and requires further studies.

It is known that MAPK, ERK, and JNK are involved in the activation of the transcription factor NF-κB, which, in turn, modulates immunological and inflammatory genes [103]. A biological molecule with a short lifespan called NO is considered a key marker of inflammatory lung diseases, including ARDS, asthma and lung fibrosis. The respiratory epithelium appears to serve as its primary source, subsequently to inducible NO synthase (iNOS) activation [104]. The production of iNOS in humans, as well as various inflammatory cytokines in the lung, are reliant on the MAPK and NF-κB signalling pathways [105]. Multiple in vitro studies have demonstrated that moxifloxacin inhibits the activation of NF-kB and MAP kinases (ERK1/2, p38, and JNK), thereby attenuating the inflammatory response induced by microbial stimuli and inflammatory mediators in various cell types (e.g., respiratory epithelial cells, monocytes) [103,104,105,106]. Moxifloxacin inhibited nitric oxide synthesis and the cytokine-induced activation of NF-κB and MAP kinases in the A549 alveolar epithelial cell line [104]; meanwhile, the expression of inflammatory mediators (IL-6, IL-8) that are dependent on NF-κB- and MAP-kinase and induced by TNF-α was inhibited in cystic fibrosis epithelial cells [106]. The activation of NF-κB and MAP kinases, as well as the release of inflammatory mediators, was also inhibited by moxifloxacin in human monocytes upon bacterial stimuli [103,107].

Quinolones eliminate bacterial cells by increasing the intracellular levels of covalent topoisomerase-cleaved DNA complexes, which serve as intermediates in these enzymes’ DNA strand-passing reactions instead of inhibiting the critical functions of type II topoisomerases. This activity elicits a high number of double-stranded breaks inside the chromosomes of treated bacteria, which induces the SOS (‘Save Our Souls’) response and, eventually, cell death [108]. Riesbeck et al. [109] also demonstrated that ciprofloxacin elicits a stress response in mammals that has a resemblance to the SOS signal response observed in bacteria. This study has shown a strong similarity between the human PBL response to topoisomerase II inhibition and the bacterial SOS response. Thus, the immunomodulatory effects of quinolones were suggested as the result of fluoroquinolones inhibiting topoisomerase II, thereby inducing a stress response in mammals that is comparable to the bacterial quinolones-induced SOS response [110]. Therefore, it is conceivable that cytosolic activation or the inhibition of NF-κB may be influenced by intra-nuclear processes involving the interaction between quinolone and topoisomerase II. A study by Zusso et al. [111] using molecular docking methods provides evidence that FQs inhibit LPS to bond with TLR4-MD-2 complex; hence, the activation of the TLR4/NF-κB signalling pathway is inhibited.

Figure 1 illustrates FQs’ mechanisms and immunomodulatory effects on ARDS (red arrow).

## 5. Evidence of FQ’s Immunomodulatory Effects Documented in Preclinical and Clinical Studies

A randomised controlled trial (RCT) conducted in Egypt revealed that treatment with 750 mg of levofloxacin once daily for 10 days affected the production of IL-10 as an anti-inflammatory cytokine and TNF-α as a pro-inflammatory cytokine, which may offer additional benefits in the treatment of respiratory tract infections irrespective of their antibacterial properties [112]. A recent in silico study provided evidence that ciprofloxacin and moxifloxacin have a potent ability to bind the main protease (M^pro^) of SARS-CoV2, indicating that fluoroquinolone can inhibit SARS-CoV2 replication [113]. According to the current guidelines and literature, fluoroquinolone has an immunomodulatory effect that is clinically beneficial for the treatment of severe pneumonia.

Several studies have reported that FQs can reduce the synthesis of pro-inflammatory cytokines. As demonstrated in an in vitro study, FQs can reduce pro-inflammatory cytokine levels in human peripheral blood mononuclear cells (PBMCs) [114]. Levofloxacin has also been shown to inhibit the secretion of TNF-α, IL-6, and IL-8 by human bronchial epithelial cells [115]. Several other studies suggest that FQs inhibit the production of IL-1 and TNF, which are pro-inflammatory cytokines [86,111,116]. Furthermore, the inhibitory effects of ciprofloxacin and levofloxacin on the NF-κB-mediated microglial inflammatory response have been reported. These effects are attained by the inhibition of lipopolysaccharide (LPS) signalling via TLR4 [107,111].

Moxifloxacin has been shown to effectively decrease the release of IL-8, IL-1b, and TNF-a, which were produced in response to Aspergillus fumigatus infection in human peripheral blood monocytes. The findings of this investigation showed that moxifloxacin inactivates the MAP-kinase ERK1/2, p38 and p65-NF-κB signalling pathways [114]. Ciprofloxacin has been demonstrated to significantly reduce the levels of TNF-α, IL-1β, and CXCL2/MIP-2a and improve the severity of lung damage and overall survival in cases of lung damage induced by LPS [32]. The study by Bailly et al. [94] revealed that both ofloxacin and norfloxacin can inhibit cytokine synthesis. Similar to ciprofloxacin, ofloxacin and grepafloxacin also inhibit the synthesis of IL-1α and IL-1β in LPS-stimulated human peripheral blood lymphocytes (hPBLs).

Another study demonstrated that the activation of TLRs on alveolar cell type II (ATII) in ARDS induces the migration of neutrophils into the epithelium, subsequently leading to the release of toxic mediators, such as proteases and ROS. FQs exhibited antioxidant activity against these conditions and suppressed pneumonia-related pulmonary inflammation [117]. Moxifloxacin has been reported to reduce neutrophil influx and pro-inflammatory cytokines levels, including keratinocytes-derived chemokine (KC), IL-1β, and IL-17A in experimental mice with lung infections induced by Streptococcus pneumoniae and *P. aeruginosa* [31]. Levofloxacin has also been shown to suppress oxidative and nitrative stress in mice models with ARDS induced by H1N1 influenza. In addition, levofloxacin demonstrated scavenging activity on neutrophil-derived ROS, resulting in a significant reduction in lung injury and an improvement in survival rates [29].

According to recent evidence, the pathogenesis of ARDS may be influenced by several immune cell types, including AMs, as mentioned before. In a healthy state, the fundamental function of AMs in tissue homeostasis is the scavenging and removal of cellular debris and apoptotic cells without inducing an inflammatory response [53]. Three typical quinolone antibiotics, ciprofloxacin, norfloxacin, and pipemidic acid, were investigated for their effects on the polarisation of macrophage RAW264.7 cells in an experimental study conducted by Lang et al. The results suggest that exposure to quinolone at environmentally relevant residual concentrations can lead to the polarisation of macrophages [118].

The immunomodulatory effect of FQ on Th cells was also documented in a study demonstrating that ciprofloxacin induced an immunomodulatory stress response in human T lymphocytes [119]. These cells are essential for humoral and cellular immunity, and the nature of the immune response is regulated by various effector Th cells (Th1, Th2, Th9, Th17, and Th22), which differentiate from naïve T cells in response to antigen stimulation [120]. The activation of T cells is facilitated by the concentration and volume of cytokine secretion in response to infection, leading to the subsequent clearance of the infection [121].

According to Kamiński et al. [122], pre-activated T cells treated with ciprofloxacin exhibited an immunosuppressed phenotype as the result of lower activation-induced ROS production, leading to the reduced expression of IL-2 and IL-4. These findings suggest that ciprofloxacin treatment could have significant implications for the management of inflammatory diseases. Furthermore, several studies in the literature demonstrate that FQs have pharmacodynamic interactions with other drugs, implying that FQs may have immunomodulatory effects, particularly following T-cell activation [123,124,125]. Findings from other studies also suggest that ciprofloxacin, at a dose of more than 20 μg/mL, is capable of counteracting cytokine production inhibition as one of cyclosporine A’s immunosuppressive effects [126]. Additionally, another study on the human leukaemia cell line (HL-60) revealed that levofloxacin-treated cells exhibited an upregulation in the mRNA expression of cytokines and chemokines (e.g., CCL2 and CXCL8) [127].

The administration of gemifloxacin resulted in the inhibition of the immune response in both the 25 mg/kg and 75 mg/kg treatment groups after a 24 h period. The study assessed the effect of gemifloxacin on the humoral component of the immune system at three different doses (25 mg/kg, 50 mg/kg, and 75 mg/kg) using heamagglutination and pneumonia plaque formation assays. The humoral immune response is produced through the synthesis of antibodies that target the specific epitope of the antigen employed to induce the immune response [85].

The summary of the role of FQs as an immunomodulator based on the findings of in vitro, in vivo and ex vivo studies both in humans and animal models is presented in Table 1.

## 6. Future Perspectives

ALI/ARDS induced by pneumonia is a heterogeneous syndrome with significant variability in pathophysiology, severity, and clinical outcomes. Infection induces an inflammatory response and initiates a complex cytokine network. Heterogeneity contributes to the inconsistent immunomodulatory effects observed in clinical studies. Immunomodulatory effects are likely to occur in the event of severe injury; however, some of the effects identified in preclinical studies are contradicting with clinical observations. A comprehensive understanding of the activation of the inflammatory cascade and the biological phenotype of ARDS patients is required in order to obtain consistent data for performing clinical trials. Phenotypically, the role of fluoroquinolones compared to other antimicrobial agents is considerably well described; however, certain features remain incompletely elucidated at present. Variations in outcomes among studies were associated with the different protocol designs and methodologies used. Although the sequence of events has not been completely understood, significant progress has been made in comprehending the specific mechanisms underlying the immunomodulatory effects of FQs, which involved the kinetic production of cytokines and chemokines, early gene transcription, and the activation or inhibition of transcription factors in cells. Therefore, further comprehensive investigation is necessary to ascertain the precise pathway that ensures FQ interacts with the cellular target topoisomerase II, as well as the subsequent impacts on the transcription mechanism.

In addition, further studies that can effectively identify the clinical characteristics of ARDS and the immunological phenotypes that are likely to respond to immunomodulatory therapy are also needed. Antibiotics administered via aerosol delivery offer higher doses that improve bacterial eradication and lower bacterial resistance. A study suggests that higher concentrations of levofloxacin administered via aerosol may provide immunomodulatory properties independent of its antimicrobial effects; however, further research on in vivo models and in patients is recommended [115].

It should be noted that not every fluoroquinolone demonstrates immunomodulatory properties. The results of several studies reported that differences in time, the frequency of administration and chemical structure may influence it. The development of study models that aim to predict desirable pharmacological properties and facilitate structural modifications of FQs will shape future generations of quinolones. The modification of the chemical structure of FQs may be necessary in order to enhance their capacity to effectively target immune function as immunomodulatory agents in addition to their ability to inhibit bacterial proliferation. The production of FQs should focus on reducing unfavourable features, including developing molecules capable of minimising off-target and drug–drug interactions. On the other side, it is critical to develop novel compounds that are capable of overcoming drug resistance. Drug resistance may be caused by protein post-translational modifications (PTMs), which are enzymatic or chemical reactions that insert covalent groups into the side chains or terminals of amino acids in proteins. Disruptive PTMs can lead to alterations in protein functions and properties that are strongly associated with the development and occurrence of numerous diseases. Targeting PTMs and associated regulatory enzymes may be highly desirable to overcome resistance to FQs and establish therapeutic prospects for various diseases, including CAP.

## 7. Conclusions

Fluoroquinolones are a class of synthetic antimicrobial agents known for their broad-spectrum activity and have been reported to exhibit immunomodulatory properties. Various pathogens, including both Gram-negative and Gram-positive bacteria, induce cytokine production through different signal transduction pathways, consequently leading to the development of CAP-associated ARDS. In vitro, in vivo, and ex vivo studies have demonstrated the modulation of innate and adaptive immune responses by FQs, which have elucidated the involvement of intracellular signal transduction pathways. According to the evidence demonstrated by these studies, the immunomodulatory activity of FQs was proven to provide indirect antibacterial effects and exhibit anti-inflammatory properties.

## Figures and Tables

**Table 1:** Immunomodulatory effects of FQs with their mechanism of action in in vitro, in vivo, ex vivo, and human models.

No	Author	Agent	Subject/Study Design/Model Disease	Outcome	Study Design	References
1	Zusso et al., 2019	Ciprofloxacin, Levofloxacin	LPS-induced primary microglia	IL-1β, TNF-α, NFkB translocation ↓	In vitro	[111]
2	Serebryakova et al., 2018	Levofloxacin	Infiltrative pulmonary tuberculosis	IL-12 in drug-sensitive tuberculosis ↓, TNFα in drug-resistant pulmonary tuberculosis ↓, IFNγ in drug-sensitive tuberculosis ↓	In vitro	[110]
3	Gupta et al., 2017	Levofloxacin	*Staphylococcus aureus*	IL-10 ↑, TNF-α, PCT, IL-1β, and IL-6 ↓	In vitro and in vivo in rats	[128]
4	Saini et al., 2015	Ciprofloxacin	*Pseudomonas aeruginosa*	MDA, NO, MIP and IL-6 ↓, IL-10 ↑	In vitro and in vivo in mice	[129]
5	Enoki et al., 2015	Levofloxacin	H1N1 influenza virus A/PR/8/34	Oxidative stress, nitrative marker, NO metabolites, ROS, and IFN-γ ↓	In vitro and in vivo in mice	[29]
6	Badari et al., 2015	Levofloxacin	TNF-α and IL-10 in the serum of pneumonic patients	TNF-α ↓ IL-10 ↓ in control, IL-10 ↑ in pneumonic patients	RCT in human	[112]
7	Müller-Redetzky et al., 2015	Moxifloxacin	*Streptococcus pneumoniae*	IL-6, IL-8, IL-1β, KC/CXCL1 ⇔	Ex vivo in human	[77]
8	Badari et al., 2014	Levofloxacin	Community-Acquired Pneumonia	TNF-α ↓ and IL-10 ↑	RCT in human	[130]
9	Beisswenger et al., 2014	Moxifloxacin	Bacterial pneumonia (*S. pneumoniae*, *Pseudomonas aeruginosa*)	KC, IL-1β, IL-17A, TNF-α-expressing cells, neutrophil influx ↓	In vivo in mice	[31]
10	Blasi et al., 2013	Levofloxacin	Chronic bronchitis	KL-6 and IL-6 ↓	Open-label, randomised study in human	[131]
11	Tsivkovskii et al., 2011	Levofloxacin	Human bronchial epithelial cells	TNF-α, IL-6, IL-8 ↓	In vitro	[115]
12	Kamiński et al., 2010	Ciprofloxacin	Pre-activated primary human T cells	TCR-induced generation ROS, IL-2 and IL-4 ↓	In vitro	[122]
13	Huang et al., 2008	Ciprofloxacin, levofloxacin, moxifloxacin	LPS	TNF-α, IL-1β, CXCL2/MIP-2α ↓ (ciprofloxacin)	In vivo in mouse	[32]
14	Calbo et al., 2008	Levofloxacin	Severe pneumococcal pneumonia	TNF-α, IL-1β, IL-6, IL-8, IL-10, and IL-1 receptor agonist ↓	Open-label, randomised study in human	[132]
15	Remund et al., 2008	Ciprofloxacin	Post-transplant bronchiolitis obliterans	TGF-β ↓ and IFN-γ ↑	In vivo in rats	[133]
16	Zhang and Ward 2007	Besifloxacin	LPS-stimulated human THP-1 monocytes (ophthalmic infections)	IL-1α, G-CSF, IL-1rα, IL-6, GM-CSF, IL-12p40, IL-1β, IL-8, IP-10, MCP-1 and MIP-1α ↓	In vitro	[87]
17	Blau et al., 2007	Moxifloxacin	Cystic fibrosis in IB3 and corrected C38 cells	IL-6, IL-8, MAPK ERK1/2, JNK, and NF-κB ↓	In vitro	[106]
18	Kitazawa et al., 2007	Levofloxacin	LPS-induced IL-1β production	pre-synthesised IL-1β, p38 ↑ IL-1β ↓	In vitro	[134]
19	Shalit et al., 2006	Moxifloxacin	Human monocytes stimulated with Aspergillus fumigatus	IL-8, IL-1β, TNF-α, MAPK ERK 1/2, p38, p65-NF-κB ↓	In vitro	[114]
20	Werber et al., 2005	Moxifloxacin	Human respiratory epithelial cell line	MAP kinase, NF-κB, NO ↓	In vitro	[104]
21	Araujo et al., 2004	Gemifloxacin	LPS-stimulated human monocytes	NF- κB, IL1α, IL-1β, IL-6, IL-10 and TNF-α ↓	In vitro	[135]
22	Gogos et al., 2004	Ciprofloxacin	Gram-negative bacteria (*Escherichia coli*, *P. aeurginosa*, *Proteus* spp., *Klebsiella pneumonia*)	IL-10 alongside the IL-10/TNF-α ratio ↑, TNF-α and IL-6 ↓	RCT in human	[136]
23	Weiss et al., 2004	Moxifloxacin	Activated human peripheral blood monocytes and THP-1 cells	IL-8, TNF-α, IL-1β, MAPK ERK 1/2, NF-κB translocation, JNK ↓	In vitro	[103]
24	Uriarte et al., 2004	Levofloxacin, moxifloxacin, gatifloxacin	HUVEC infected with *Chlamydophila pneumoniae* or stimulated with TNF-α	Neutrophil and monocyte TEM ↓ IL-8 ↓ (MOX and GTFX) MCP-1 ↓ (MOX)	In vitro	[137]
25	Shalit et al., 2002	Moxifloxacin	*Candida albicans*, cyclophosphamide	TNF-α, KC/CXCL1 ↓, IL-2, IL-10, IFN-γ ⇔	In vivo in mice	[33]
26	König et al., 2002	Moxifloxacin	Staphylococcal superantigen-induced apoptosis	Fas, FasL, and TNF- RI ↓	In vitro	[138]
27	Araujo et al., 2002	Moxifloxacin	LPS-stimulated monocytes	IL-1α, IL-1β, IL-6, IL-10, IL-12 (p70), TNF-α ↓	In vitro	[107]
28	Ono et al., 2000	Grepafloxacin	LPS-stimulated human peripheral blood cells	IL-2 ↑, TNF-α, IL-8, IL-1α, and IL-1β ↓	In vitro	[139]
29	Khan et al., 1998	Trovafloxacin	Human monocytes stimulated by LPS or *S. aureus* heat-killed cells	IL-1α, IL-1β, IL-6, IL-10, GM-CSF, and TNF-α ↓	In vitro	[140]
30	Nwariak FE et al., 1997	Ciprofloxacin	*P. aeruginosa*	TNF response ↓	In vitro samples obtained from rabbits	[141]
31	Riesbek et al., 1994	Ciprofloxacin	Lymphocytes incubated with cyclosporine	IFN-γ, IL-2 ↑	In vitro	[126]
32	Bailly et al., 1990	Ciprofloxacin, Ofloxacin, Grepafloxacin	in LPS-stimulated hPBL	TNF, IL-1α, IL-1β ↓	In vitro	[94]

↓: Decreased/downregulated/inhibited; ↑: increase/upregulated/stimulated; ⇔: no significant difference; CXCL: C-X-C motif chemokine ligand; GM-CSF: granulocyte–macrophage colony-stimulating factor; GTFX: gatifloxacin; IFN: interferon; IL: interleukin; KC: keratinocytes-derived chemokine; KL: Krebs von den Lungen; LPS: lipopolysaccharide; MAPK: MAP-Kinases; MDA: malondialdehyde; MIP: macrophage inflammatory protein; MOX: moxifloxacin; NF-κB: NF-kappaB; NO: nitric oxide; *P. aeruginosa*: *Pseudomonas aeruginosa*; ROS: reactive oxygen species; S.: *Streptococcus*; TGF-β: transforming growth factor-β; TNF-α: tumour necrosis factor-α.

## Data Availability

Not applicable.

## Abbreviations

ALI	Acute lung injury
AMs	Alveolar macrophages
AP-1	Activator protein-1
ARDS	Acute respiratory distress syndrome
ATII	Alveolar cell type II
BAL	Bronchoalveolar lavage
cAMP	Cyclic adenosine monophosphate
CAP	Community-acquired pneumonia
cGMP	Cyclic guanosine monophosphate
CREB	cAMP response element-binding protein
CRP	C-reactive protein
DAMPs	Damage-associated molecular pattern molecules
ERK	Extracellular signal-regulated kinases
FQs	Fluoroquinolones
GM-CSF	Granulocyte–macrophage colony-stimulating factor
hPBL	Human peripheral blood lymphocytes
IFN	Interferon
IL	Interleukin
iNOS	Inducible NO synthase
IκB	Inhibitory κappa B
JNK	c-Jun N-terminal kinases
KC	Keratinocytes-derived chemokine
LPS	Lipopolysaccharide
MAMPs	Microbe-associated molecular patterns
MAPK	Mitogen-activated protein kinase
MCP-1	Monocyte chemotactic protein
MIP-1α	Macrophage inflammatory protein 1α
M^pro^	Main protease
NET	Neutrophil extracellular trap
NF-κB	Nuclear factor κappa-B
NO	Nitric oxide
PAMPs	Pathogen-associated molecular patterns
PBMCs	Peripheral blood mononuclear cells
PDEs	Phosphodiesterases
PKA	Protein kinase A
PRRs	Pattern recognition receptors
RBCs	Red blood cells
RCT	Randomised controlled trial
ROS	Reactive oxygen species
SARS-CoV-2	Severe acute respiratory syndrome coronavirus 2
TAK1	Transforming growth factor-activated kinase 1
TLR	Toll-like receptor
TNF	Tumour necrosis factor
